# Stable Isotope Evidence for Late Medieval (14th–15th C) Origins of the Eastern Baltic Cod (*Gadus morhua*) Fishery

**DOI:** 10.1371/journal.pone.0027568

**Published:** 2011-11-16

**Authors:** David C. Orton, Daniel Makowiecki, Tessa de Roo, Cluny Johnstone, Jennifer Harland, Leif Jonsson, Dirk Heinrich, Inge Bødker Enghoff, Lembi Lõugas, Wim Van Neer, Anton Ervynck, Anne Karin Hufthammer, Colin Amundsen, Andrew K. G. Jones, Alison Locker, Sheila Hamilton-Dyer, Peter Pope, Brian R. MacKenzie, Michael Richards, Tamsin C. O'Connell, James H. Barrett

**Affiliations:** 1 McDonald Institute for Archaeological Research, University of Cambridge, Cambridge, United Kingdom; 2 Institute of Archaeology, Nicolaus Copernicus University, Toruń, Poland; 3 Department of Archaeology and Anthropology, University of Cambridge, Cambridge, United Kingdom; 4 Department of Archaeology, University of York, York, United Kingdom; 5 Museum of Natural History, Gothenburg, Sweden; 6 Zoological Institute, Christian-Albrechts-Universität zu Kiel, Kiel, Germany; 7 Natural History Museum of Denmark (Zoological Museum), University of Copenhagen, Copenhagen, Denmark; 8 Department of Archaeobiology (Institute of History), University of Tallinn, Tallinn, Estonia; 9 Royal Belgian Institute of Natural Sciences, Brussels, Belgium; 10 Laboratory of Animal Biodiversity and Systematics, Katholieke Universiteit Leuven, Leuven, Belgium; 11 Flemish Heritage Institute, Brussels, Belgium; 12 Bergen Museum, University of Bergen, Bergen, Norway; 13 Department of Archaeology and Social Anthropology, University of Tromsø, Tromsø, Norway; 14 York Archaeological Trust, York, United Kingdom; 15 Escaldes-Engordany, Andorra; 16 Southampton, United Kingdom; 17 Department of Archaeology, Memorial University of Newfoundland, St. John's, Newfoundland, Canada; 18 National Institute for Aquatic Resources, Technical University of Denmark, Charlottenlund, Denmark; 19 Department of Anthropology, University of British Columbia, Vancouver, British Columbia, Canada; 20 Department of Human Evolution, Max Planck Institute for Evolutionary Anthropology, Leipzig, Germany; National Institute of Water & Atmospheric Research, New Zealand

## Abstract

Although recent historical ecology studies have extended quantitative knowledge of eastern Baltic cod (*Gadus morhua*) exploitation back as far as the 16th century, the historical origin of the modern fishery remains obscure. Widespread archaeological evidence for cod consumption around the eastern Baltic littoral emerges around the 13th century, three centuries before systematic documentation, but it is not clear whether this represents (1) development of a substantial eastern Baltic cod fishery, or (2) large-scale importation of preserved cod from elsewhere. To distinguish between these hypotheses we use stable carbon and nitrogen isotope analysis to determine likely catch regions of 74 cod vertebrae and cleithra from 19 Baltic archaeological sites dated from the 8th to the 16th centuries. δ^13^C and δ^15^N signatures for six possible catch regions were established using a larger sample of archaeological cod cranial bones (n = 249). The data strongly support the second hypothesis, revealing widespread importation of cod during the 13th to 14th centuries, most of it probably from Arctic Norway. By the 15th century, however, eastern Baltic cod dominate within our sample, indicating the development of a substantial late medieval fishery. Potential human impact on cod stocks in the eastern Baltic must thus be taken into account for at least the last 600 years.

## Introduction

### Overview and hypotheses

Recent studies of eastern Baltic cod (*Gadus morhua*) populations - particularly within the framework of the *History of Marine Animal Populations* project - stress the importance of time depth for understanding impacts of interactions between fishing mortality and other factors on stocks [1]–[6]. The long-term history of Baltic cod fisheries remains poorly understood, however, and this paper contributes by establishing the historical origin of commercial cod fishing in the eastern Baltic.

Tax records document commercial cod fishing in various parts of the eastern Baltic as early as the 1550s [7], but archaeological evidence for consumption of cod at multiple medieval settlements around the eastern Baltic littoral starts considerably earlier, in the mid-13th century. The fish may not have been caught locally, however; preserved cod are known from historical sources to have been imported from Norway to western Baltic settlements during the medieval period [8] and may also have been traded further to the east. Two alternative hypotheses can therefore be proposed:

1. The eastern Baltic cod fishery emerged in the 13th century, predating its systematic historical documentation by as much as three centuries.
2. Cod consumed around the eastern Baltic littoral during the medieval period were overwhelmingly imported, with a local eastern Baltic fishery developing subsequently.

**Figure 1:**
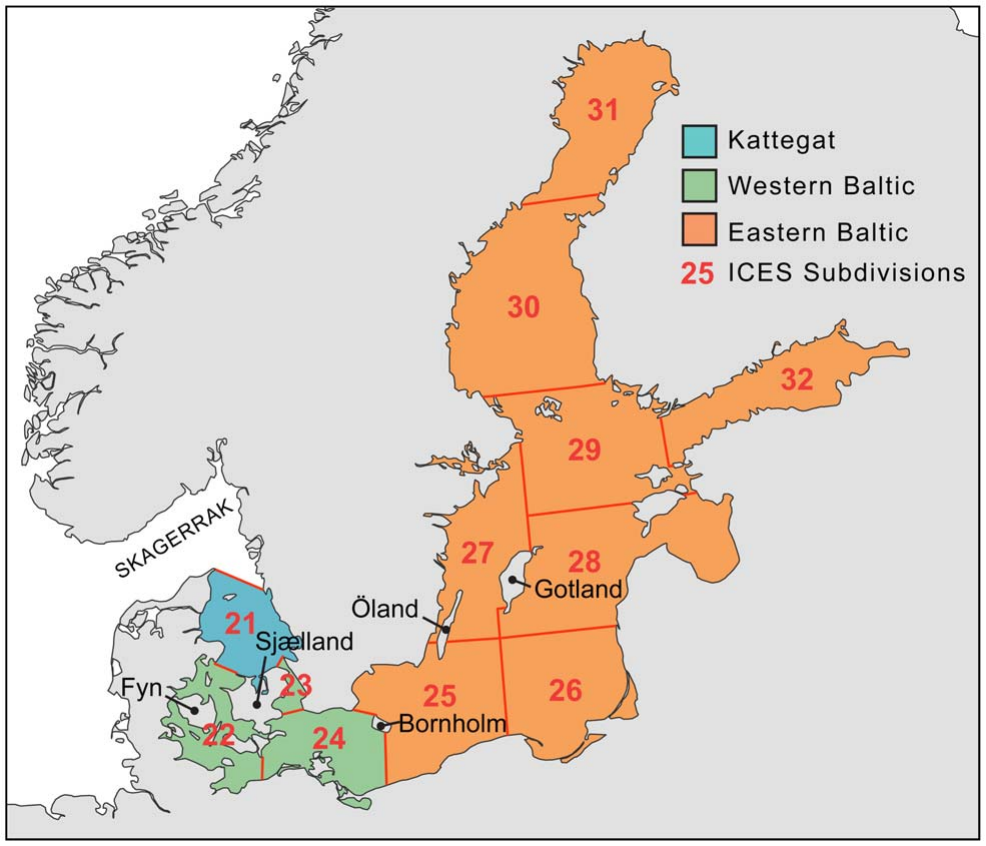
Map showing definitions of eastern Baltic, western Baltic, and Kattegat. Individual ICES subdivisions and major islands mentioned in the text are also marked.

We use stable carbon and nitrogen isotopic signatures to test these hypotheses by assigning likely provenances to 13th- to 16th-century cod bones recovered from archaeological sites around the eastern Baltic littoral (defined as east of Bornholm; figure 1). Samples from western Baltic settlements are also analysed, to provide a comparative case where both earlier medieval cod fishing and cod trade have been documented by past research [9], [10].

### Background

Archaeology is an increasingly valuable methodology in global efforts to understand long-term trends in the utilization and alteration of marine ecosystems by humans [11]. The archaeological study of changing economic patterns – such as the expanding demand of growing, often urban, populations – has converged with environmental history as it reveals the relentless extraction of resources from both local and distant waters [12]. Yet the relationship between economic and ecological pressure is not simple. Responses to increased demand can vary between extensification (seeking ever more distant resources) and intensification (more labour-intensive exploitation of comparatively local waters) in complex ways, all mediated by the cultural filters of politics, tradition and belief. The present study seeks to illuminate these distinct yet interrelated variables by tracing the geographical origin of cod consumed in the Baltic Sea region, from the 8th century until the emergence of systematic catch statistics in the 16th century. In so doing it informs the economic and environmental ‘history’ of Europe, while also demonstrating the need to consider cultural factors that may lead to unexpected and counter-intuitive twists within an overall human trend of using increasingly distant resources [13].

Baltic cod in the area to the east of Bornholm (ICES subdivisions 25–32) comprise a distinct population from that in the western Baltic and Kattegat (ICES subdivisions 21–24) [14], and are sometimes considered a different subspecies (*G. morhua callarias* as opposed to *G. morhua morhua*) [15], [16]. ICES (International Council for the Exploration of the Sea) subdivisions are universally recognized, unambiguous divisions that in this case are also coterminous with the approximate boundary between populations. Prehistoric exploitation of cod in both areas is attested by osteological remains from archaeological sites [17]–[23]. It virtually ceases in the Baltic proper with the end of the Neolithic period (c. 2500 BC), although cod do continue to be found at some sites on the Kattegat during the Bronze Age. Cod remains then reappear in substantial quantities in mid 1st millennium AD archaeological contexts on the western Baltic islands of Fyn and Sjælland, on Bornholm, and on the eastern Baltic island of Öland [9], [24]–[27]. They are common during the Viking Age (8th–11th C) around the western Baltic, where their frequency increases into the subsequent medieval period, alongside the first evidence for deep sea fishing in the region [9]. Viking Age cod are also found at the trading centre of Visby on Gotland [28].

Conversely, around the eastern Baltic littoral - apart from the islands - evidence for cod consumption remains rare until around the 13th century [29]. In eastern Sweden, cod specimens are virtually absent from a very large studied assemblage at 8th– 10th-century Birka [30], are noted as present in 12th-century layers at Sigtuna [31], but are the most common species amongst fish remains from 13th- to 14th-century Uppsala [32]. In Estonia, only freshwater species are found at Viking Age coastal settlements, but cod bones make up over 20% of fish remains from 13th-century Tallinn and are present in smaller proportions (2–4%) at 14th-century Pärnu and as far inland as 13th–14th-century Tartu [29], [30], [33]. In Poland, only four cod bones have been identified out of around 20,000 studied specimens from five 9th to 12th-century coastal settlements, while in the 13th–16th centuries the species is represented in almost every studied coastal assemblage (and even at some settlements more than 50 km inland) in proportions ranging from a few percent to around half of identified fish remains [34].

The relative explosion in cod consumption during the 13th to 14th centuries coincides with major historical developments in the region. This period saw the interconnection of much of the eastern Baltic littoral with wider political and economic systems: German and Danish crusaders established settlements in Estonia and Latvia; most of the Polish coast came under the political influence of the Teutonic Order; and the Hanseatic League extended its economic activities throughout the Baltic and beyond. Evidence for cod consumption during the 13th–14th centuries comes precisely from the sites most connected with these developments, including colonial towns such as Tallinn and Pärnu, Polish coastal strongholds that had come under Germanic influence - e.g. Gdańsk, Kołobrzeg - and Teutonic Order castles such as Mała Nieszawka (Poland).

Medieval evidence for cod consumption at *inland* settlements in the eastern Baltic region indicates that fish were preserved and traded during this period, at least on a local level [9], [30], [35]. Specimens from 12th- to 14th-century sites are often larger than either modern or prehistoric Baltic cod, suggesting that preserved fish were imported from the Kattegat or beyond [9], [30], [34]. This is supported by the anatomical distribution of specimens: cod cranial elements (typically removed before preservation and transport [36])are sometimes (a) much rarer and (b) from smaller fish than vertebrae found at the same sites [31], [32].

It is historically plausible that cod consumed at medieval Baltic settlements were overwhelmingly imported. Export-driven cod fisheries are known to have developed in Arctic Norway by the 11th to 12th centuries [37], and the Hanseatic League came to dominate trade in stockfish - decapitated and dried cod - during the 13th to 14th centuries [38], [39]. This can be seen as an early example of food globalisation, with consumers increasingly detached from producers as expanding markets pushed resource exploitation well beyond local ecosystems [40]. Stockfish were typically shipped from Bergen to Lübeck during this period [8] and may have been traded on to Hanse ports in the eastern Baltic. If the cod consumed at 13th–14th century settlements in the eastern Baltic can be demonstrated to have been imported, this would confirm the integration of those settlements into a network of trade in fish that stretched at least from northern Norway to London and from Iceland to Tartu.

Historical records indicate the existence of taxable commercial cod fisheries off eastern Sweden and south-west Finland (ICES subdivisions 27 and 29) by the 1550s, and in the southern Baltic (ICES subdivision 25) by the start of the 17th century [7], [41]. A 16th-century origin for eastern Baltic fisheries would coincide with challenges to Hanseatic dominance of the stockfish trade and declining fish prices [8], [38]. On the other hand, absence of historical records from preceding centuries cannot be taken as evidence that eastern Baltic cod fisheries did not exist.

In order to distinguish between local (Baltic) fishing and importation of preserved cod from, for example, the North or Barents Seas, it is necessary to assign approximate provenances (i.e. catch regions) to cod bone specimens recovered from archaeological sites. We do so using stable carbon and nitrogen isotopic signatures, following a new methodology [10] recently applied to archaeological sites around the North Sea [12].

### Stable Isotope Analysis

Carbon and nitrogen isotopic values (δ^13^C and δ^15^N) in fish tissues are likely to vary from region to region since they are known to change with temperature, salinity, nutrient loading, and trophic level [42]–[46]. They have been shown to differ between modern cod populations [47]. Since cod has well-understood migration patterns in which many populations remain close to their spawning grounds [15], [48]–[51], isotopic signatures can in principle be used to assign individual specimens to likely catch regions. Well-documented increases in nutrient loading during the last hundred years, however - especially in the North and Baltic Sea littorals [52]–[57] - prevent extrapolation of modern isotopic data to historical periods. Instead, isotopic signatures for potential catch regions must be derived from archaeological material.

**Figure 2:**
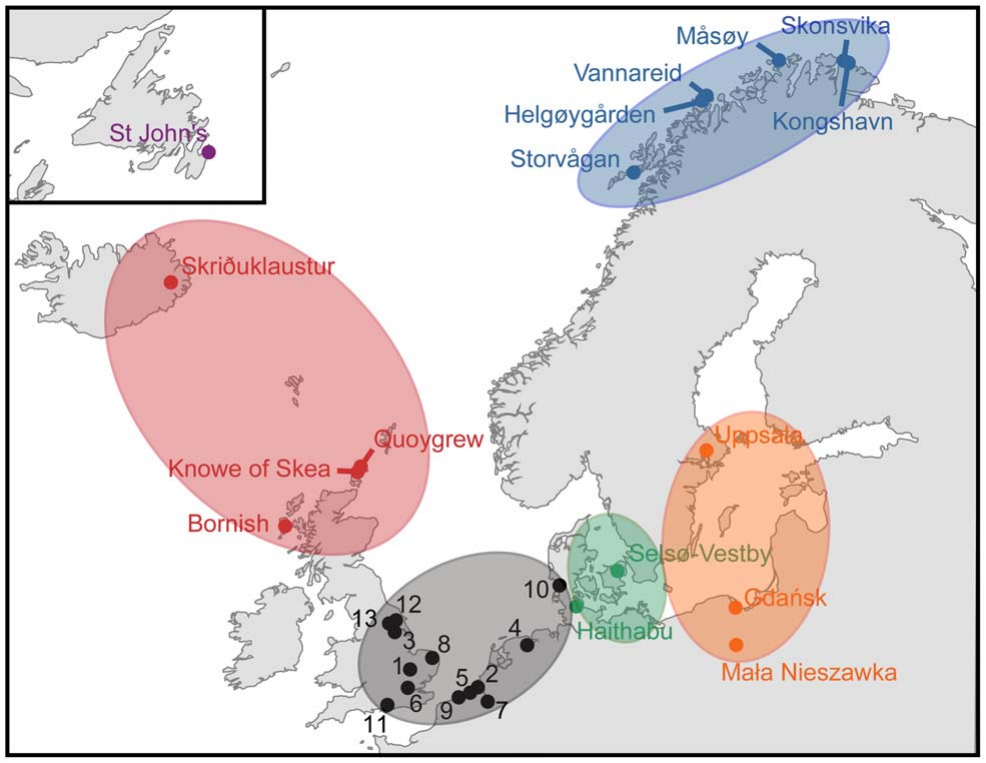
Sources of archaeological control specimens. Settlements are coloured by analytical region (see figure 3). Key to southern North Sea settlements: 1-Cambridge; 2-Castle Sint-Maartensdijk; 3-Flixborough; 4-Groningen; 5-Heist; 6-London; 7-Mechelen; 8-Norwich; 9-Raversijde; 10 - Ribe; 11-Southampton; 12-Wharram Percy; 13-York. Note that some large settlements provided samples from multiple archaeological sites.

Our approach involves two steps [10]. Firstly, collagen from archaeological cod *cranial* bones (‘control specimens’) was used to identify isotopic (δ^13^C and δ^15^N) signatures for six potential catch regions (eastern Baltic, Kattegat/western Baltic, southern North Sea, northeast Atlantic, northwest Atlantic, Arctic Norway; figure 2). Cranial bones are likely to represent individuals caught relatively locally since - before the development of refrigeration - cod were typically decapitated and dried for long-range transport or trade.

Secondly, the same isotopic values are measured for *postcranial* bones (‘target specimens’), specifically vertebrae and cleithra, recovered from Viking Age and medieval sites around the Baltic. Since these elements were typically included in traditional preserved fish products such as stockfish [36], [58], target specimens may derive either from local catches or from imported fish. Comparison with the regional isotopic signatures established in the first step allows individual target specimens to be assigned to their most likely catch regions.

This approach has previously been used successfully to provenance cod remains from medieval sites around the North Sea littoral [12]. The same methodology is applied here, with minor modifications to incorporate control and target samples from cod with estimated total lengths (TL) greater than 1 m (see materials and methods section, below). Such large fish - extremely rare in both modern and prehistoric Baltic populations - are common at many Baltic medieval sites and are often assumed to represent imports; excluding them from the study would therefore prejudice the results. Since fish size may influence isotopic values, all stages of analysis are carried out separately for specimens from cod with estimated lengths above and below 1000 mm (see materials and methods). The main control sample (171 cod with estimated TL 500–1000 mm) is identical to that used by Barrett *et al.* [12], and is supplemented here by 78 specimens with estimated TL>1000 mm (table S1).

## Results

### Control samples

**Figure 3:**
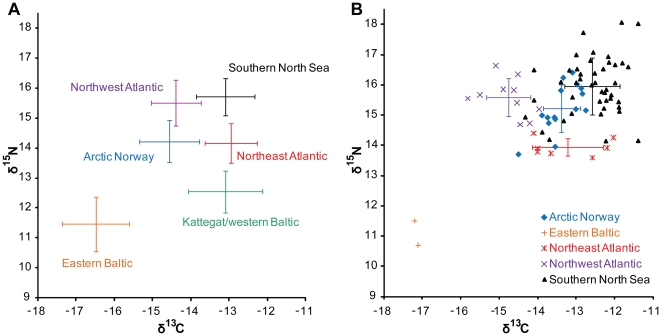
δ^13^C and δ^15^N for control specimens from each geographical region. A: means and 1σ ranges for cod in the 500–1000 mm size class (data from [12]); B: new data for cod in the >1000 mm size class.

Group means and 1σ confidence intervals for carbon and nitrogen isotopic values from the 500–1000 mm control sample are shown in figure 3a. The eastern Baltic sample is particularly clearly separated from other regions, due in part to depleted δ^13^C values that may reflect the low salinity of the Baltic proper. By contrast the Kattegat/western Baltic sample falls closer to non-Baltic regions, with a large standard deviation in δ^13^C probably resulting from marked spatial and temporal variability in salinity [59], [60], possibly compounded by fish migration within the region [61].

Since regional isotopic signatures might conceivably have changed over time, it was necessary to assess possible temporal trends within the control data for each region using ANOVA (see materials and methods). For the 500–1000 mm control group, the only significant difference between time periods was for the Eastern Baltic, where a decrease was observed in δ^13^C between 13th/14th-century (n = 9) and 15th/16th-century (n = 21) specimens (F(df 1, 28) = 15.58, *p* = 0.0005; mean difference = 1.1‰). The scale of this decrease is insufficient to obscure differences between regions, however, with the eastern Baltic remaining clearly separated from other regions when δ^13^C and δ^15^N are taken together (figure 3a). Raw data and graphic temporal comparisons for all regions are published in a previous study [12].

Target specimens within this size range were attributed to probable source regions using discriminant function analysis (DFA). The northeast Atlantic and Arctic Norwegian regions were grouped into a single source (‘northern’) for this purpose. Historical records indicate that dried cod produced both in Norway and in the Scandinavian north-east Atlantic colonies were traded via Bergen during the Middle Ages [62], so would have represented a single source of imports from the point of view of Baltic consumers. Taking the conservative view that cod from the north-west Atlantic (including the Grand Banks) are unlikely to have been traded to Europe before Zuan Caboto's transatlantic expedition in 1497, DFA was performed both excluding and including the control data from Newfoundland, for applicability to earlier and later target specimens respectively. This matches the approach taken in a recent study of cod imports to the southern North Sea littoral [12]. Reclassification success rates range from 73% to 93% including Newfoundland, and from 80% to 93% excluding it. Crucially for the present study, the success rate for the eastern Baltic is 93% in all cases, and that for the Kattegat/western Baltic is 80%.

Results for the >1000 mm sample are plotted in figure 3b. The relative positions of the southern North Sea, northeast Atlantic, and northwest Atlantic groups are broadly similar to those for smaller fish, but significant elevation of both δ^13^C and δ^15^N amongst large Arctic Norwegian cod results in substantial overlap with the southern North Sea group. This implies a stronger relationship between fish size (and hence age) and trophic level amongst cod caught off northern Norway (likely primarily to be migratory Barents Sea cod [10]) than amongst other populations. For the present study, the implication is that imports of very large cod (TL>1000 mm) cannot be reliably attributed to specific non-Baltic regions using stable isotopes.

No cod cranial bones in the >1000 mm size category were obtained from the Kattegat/western Baltic region, and only two could be sampled from the eastern Baltic. This is not a sample selection issue but simply reflects the near-total absence of such specimens in the archaeological record: as with both modern populations and prehistoric data from the region [5],[17], cod measuring more than 1 m appear to have been extremely rare in the Baltic Sea during the Viking Age and medieval periods. The two such specimens that were obtained, however, fit very well with the eastern Baltic data for smaller fish, each falling within one standard deviation of the group means for both δ^13^C and δ^15^N.

Given the very limited Baltic data and the overlap between non-Baltic regions, DFA is clearly not applicable for the >1000 mm sample. Instead, all non-Baltic control regions were grouped. Target specimens were then evaluated in terms of Mahalanobis distance (*D* ^2^) from the resulting centroid. Targets falling within a 95% confidence zone were assumed to be either from non-Baltic sources or the Kattegat/western Baltic. Those falling outside this zone were considered indeterminate but compared with the eastern Baltic data qualitatively in the discussion. The two eastern Baltic control specimens themselves fall well outside the confidence zone - *p*<0.0001 in both cases - while only one non-Baltic control is a significant outlier (specimen 944: *p* = 0.0175). Regional temporal comparisons were not carried out for this size class due to the pooling of all non-Baltic regions and the limited sample from the eastern Baltic. The non-Baltic group includes samples spanning the 10th–18th centuries, but remains clearly distinct from the available 14th/15th-century eastern Baltic controls.

### Target samples

**Figure 4:**
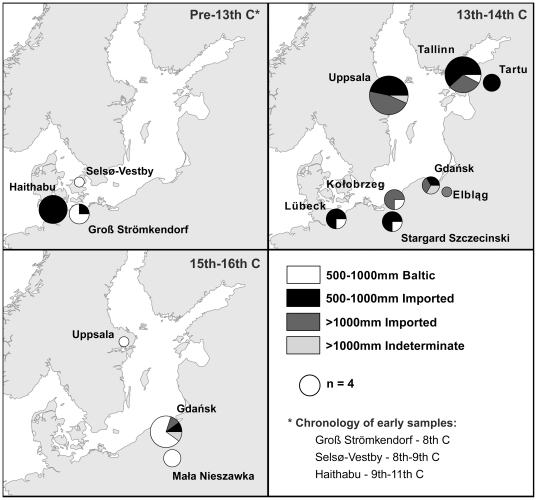
Overview of isotopic evidence for imported versus Baltic cod at archaeological sites. Note that transport within the Baltic region is not made apparent here but does seem to have taken place in a few cases. Samples with date ranges spanning the 14th–15th centuries were assigned to chronological group according to greatest overlap, favouring the later period where overlap with each was equal.

The target sample is composed of 56 vertebrae and 18 cleithra from 20 Baltic sites dated to the 8th to 16th centuries (table 1). Of these, 9 appeared in a pilot study [10] and the remainder are published here for the first time. δ^13^C and δ^15^N values have previously been published for 6 additional cod vertebrae from the German Viking Age and medieval sites of Haithabu and Schleswig as part of an ecosystem study [63] but are not used here due to inter-laboratory differences in sample preparation. Isotopic values, dating, and likely provenance are provided for individual specimens in tables 2 and 3 (TL 500–1000 mm) and table 4 (TL>1000 mm), while figure 4 gives an overview of change through time.

**Table 1:** Summary of target (postcranial) samples from the Baltic region by archaeological site.

Region	Site	Country	Lat. (°N)	Long. (°E)	Date (AD)	Cleithra	Vertebrae
***Kattegat/western Baltic***	Groß Strömkendorf	Germany	53.96	11.49	8thC		4
	Haithabu Harbour	Germany	54.49	9.57	9th–11th C	4	2
	Haithabu Settlement Area	Germany	54.49	9.57	9th–11th C		2
	Lübeck, Dr Julius-Leber-Straße	Germany	53.87	10.69	14th C		3
	Lübeck, Fleischhauerstraße 64–72	Germany	53.87	10.69	14th C	1	
	Selsø-Vestby	Denmark	55.74	12.01	8th–9th C	1	
	Stargard Szczeciński 11a	Poland	53.34	15.05	L.13th–E.15th C		4
***Eastern Baltic***	Elbląg	Poland	54.16	19.40	13th C		1
	Gdańsk, Granary Island	Poland	54.35	18.66	L.14th–16th C	4	5
	Gdańsk, Green Gate	Poland	54.35	18.66	15th–16th C	1	
	Gdańsk, Olejarna Street 2	Poland	54.36	18.66	1255–1295		3
	Kołobrzeg, Armii Krajowej St. 19	Poland	54.18	15.57	12th–14th C		2
	Kołobrzeg, Gierczak Street	Poland	54.17	15.58	L.14th C	1	1
	Mała Nieszawka	Poland	52.99	18.55	14th–15th C	3	
	Tallinn, 10 Sauna St	Estonia	59.44	24.75	L.13th–E.14th C		8
	Tallinn, 10 Viru St	Estonia	59.44	24.75	M.13th–L.14th C	1	2
	Tallinn, 4 Rahukohtu St	Estonia	59.44	24.74	M.13th C		2
	Tartu, Vanemuise St.	Estonia	58.37	26.72	L.13th–E.14th C	1	2
	Uppsala, Kransen Block	Sweden	59.86	17.64	13th C; 15th C	1	15
***Total samples***						18	56

**Table 2:** Isotopic results for smaller (estimated TL 500–1000 m) target specimens from the Kattegat/western Baltic.

Site	ID	Date (AD)	Element	Est. length (mm)	δ^13^C	δ^15^N	C∶N	Predicted provenance	Prob.	Status
**Groß Strömkendorf**	**197**	**8th C**	**Vertebra**	**500–800**	**−12.63**	**12.97**	**3.4**	**Kattegat/western Baltic**	**0.89**	**Local**
Groß Strömkendorf	323	8th C	Vertebra	500–800	−14.22	13.46	3.3	Northern	0.73	Import
**Groß Strömkendorf**	**324**	**8th C**	**Vertebra**	**500–800**	**−15.52**	**12.82**	**3.6**	**Eastern Baltic**	**0.60**	**Cross-Baltic**
**Groß Strömkendorf**	**325**	**8th C**	**Vertebra**	**500–800**	**−9.96**	**11.10**	**3.3**	**Kattegat/western Baltic**	**1.00**	**Local**
**Selsø-Vestby**	**224**	**8th–9th C**	**Cleithrum**	**500–800**	**−12.57**	**12.06**	**3.3**	**Kattegat/western Baltic**	**0.99**	**Local**
Haithabu Harbour	315	9th–11th C	Vertebra	800–1000	−14.98	13.97	3.4	Northern	0.94	Import
Haithabu Harbour	316	9th–11th C	Vertebra	800–1000	−14.72	15.07	3.3	Northern	0.56	Import
Haithabu Harbour	317	9th–11th C	Cleithrum	800–1000	−14.23	14.36	3.4	Northern	0.87	Import
Haithabu Harbour	318	9th–11th C	Cleithrum	800–1000	−14.49	14.87	3.3	Northern	0.67	Import
Haithabu Harbour	319	9th–11th C	Cleithrum	800–1000	−11.86	15.86	3.1	Southern North sea	0.96	Import
Haithabu Harbour	320	9th–11th C	Cleithrum	800–1000	−14.81	14.59	3.3	Northern	0.84	Import
Haithabu Settlement area	826	9th–11th C	Vertebra	800–1000	−14.70	13.20	3.3	Northern	0.63	Import
Haithabu Settlement area	828	9th–11th C	Vertebra	800–1000	−13.90	14.30	3.3	Northern	0.86	Import
Stargard Szczeciński 11a	1159	L.13th–E.15th C	Vertebra	800–1000	−13.10	15.00	3.3	Southern North sea	0.57	Import
Stargard Szczeciński 11a	1160	L.13th–E.15th C	Vertebra	800–1000	−14.60	13.60	3.3	Northern	0.85	Import
**Stargard Szczeciński 11a**	**1161**	**L.13th–E.15th C**	**Vertebra**	**500–800**	**−16.90**	**12.20**	**3.6**	**Eastern Baltic**	**1.00**	**Cross-Baltic**
Stargard Szczeciński 11a	1162	L.13th–E.15th C	Vertebra	800–1000	−15.80	14.20	3.6	Northern	0.95	Import
**Lübeck, Fleischhauerstraße 64–72**	**815**	**14th C**	**Cleithrum**	**500–800**	**−14.80**	**11.30**	**3.3**	**Eastern Baltic**	**0.76**	**Cross-Baltic**
Lübeck, Dr Julius-Leber-Straße	816	14th C	Vertebra	800–1000	−12.30	14.80	3.2	Southern North sea	0.52	Import
Lübeck, Dr Julius-Leber-Straße	818	14th C	Vertebra	800–1000	−14.60	14.80	3.3	Northern	0.72	Import
Lübeck, Dr Julius-Leber-Straße	819	14th C	Vertebra	800–1000	−14.00	14.90	3.4	Northern	0.60	Import

Provenances are as predicted by DFA, along with probability of membership of the relevant geographical group. Specimens in bold face are those which appear to have been caught in the Baltic.

**Table 3:** Isotopic results for smaller (estimated TL 500–1000 m) target specimens from the Eastern Baltic.

Site	ID	Date (AD)	Element	Est. length (mm)	δ^13^C	δ^15^N	C∶N	Predicted provenance	Prob.	Status
Tallinn, 4 Rahukohtu St	491	1225–1250	Vertebra	800–1000	−14.41	14.31	3.3	Northern	0.89	Import
Tallinn, 4 Rahukohtu St	492	1225–1250	Vertebra	500–800	−14.61	15.12	3.4	Northern	0.51	Import
Uppsala	327	L. 13^th^ C	Vertebra	800–1000	−15.44	13.23	3.3	Northern	0.65	Import
Uppsala	332	L. 13^th^ C	Vertebra	800–1000	−14.19	15.25	3.4	Southern North sea	0.62	Import
Uppsala	334	L. 13^th^ C	Vertebra	800–1000	−14.03	15.43	3.1	Southern North sea	0.75	Import
Uppsala	335	L. 13^th^ C	Vertebra	800–1000	−14.74	13.50	3.4	Northern	0.83	Import
Uppsala	336	L. 13^th^ C	Vertebra	800–1000	−14.71	13.86	3.3	Northern	0.92	Import
Uppsala	337	L. 13th C	Vertebra	800–1000	−14.65	14.27	3.4	Northern	0.91	Import
Uppsala	338	L. 13th C	Vertebra	800–1000	−13.16	14.06	3.3	Northern	0.82	Import
Tallinn, 10 Sauna St	480	L.13th–E.14th C	Vertebra	800–1000	−14.35	16.08	3.3	Southern North sea	0.94	Import
Tallinn, 10 Sauna St	482	L.13th–E.14th C	Vertebra	800–1000	−14.33	14.41	3.1	Northern	0.86	Import
Tallinn, 10 Sauna St	483	L.13th–E.14th C	Vertebra	800–1000	−11.51	15.87	3.2	Southern North sea	0.97	Import
**Tallinn, 10 Sauna St**	**485**	**L.13th–E.14th C**	**Vertebra**	**800–1000**	**−13.62**	**13.15**	**3.2**	**Kattegat/western Baltic**	**0.63**	**Cross-Baltic**
Tallinn, 10 Sauna St	486	L.13th–E.14th C	Vertebra	500–800	−13.52	14.62	3.2	Northern	0.72	Import
Tartu, Vanemuise St.	493	L.13th–E.14th C	Vertebra	800–1000	−14.18	15.73	3.2	Southern North sea	0.87	Import
Tartu, Vanemuise St.	494	L.13th–E.14th C	Vertebra	500–800	−14.57	14.27	3.3	Northern	0.91	Import
Tartu, Vanemuise St.	495	L.13th–E.14th C	Cleithrum	500–800	−14.86	14.31	3.2	Northern	0.91	Import
Gdańsk, Olejarna Street 2	469	1350–1400	Vertebra	800–1000	−14.73	14.75	3.3	Northern	0.76	Import
**Kołobrzeg, Gierczak Street**	**476**	**1350–1400**	**Cleithrum**	**800–1000**	**−15.55**	**11.39**	**3.1**	**Eastern Baltic**	**0.97**	**Local**
Tallinn, 10 Viru St	487	1350–1400	Cleithrum	800–1000	−11.68	15.11	3.1	Southern North sea	0.77	Import
Tallinn, 10 Viru St	488	1350–1400	Vertebra	500–800	−13.00	14.39	3.2	Northern	0.77	Import
**Gdańsk, Granary Island**	**1137**	**1350–1450**	**Vertebra**	**800–1000**	**−16.20**	**9.40**	**3.1**	**Eastern Baltic**	**1.00**	**Local**
**Gdańsk, Granary Island**	**1138**	**1350–1450**	**Cleithrum**	**500–800**	**−17.70**	**11.30**	**3.4**	**Eastern Baltic**	**1.00**	**Local**
**Gdańsk, Granary Island**	**1154**	**1350–1450**	**Cleithrum**	**800–1000**	**−17.90**	**10.30**	**3.3**	**Eastern Baltic**	**1.00**	**Local**
**Mała Nieszawka**	**215**	**14th–15th C**	**Cleithrum**	**500–800**	**−18.03**	**11.06**	**3.6**	**Eastern Baltic**	**1.00**	**Local**
**Mała Nieszawka**	**216**	**14th–15th C**	**Cleithrum**	**500–800**	**−16.53**	**10.87**	**3.2**	**Eastern Baltic**	**1.00**	**Local**
**Mała Nieszawka**	**217**	**14th–15th C**	**Cleithrum**	**500–800**	**−15.69**	**11.86**	**3.2**	**Eastern Baltic**	**0.97**	**Local**
**Gdańsk, Granary Island**	**1150**	**14th–15th C**	**Vertebra**	**800–1000**	**−17.00**	**10.80**	**3.6**	**Eastern Baltic**	**1.00**	**Local**
**Uppsala**	**466**	**15th C**	**Cleithrum**	**800–1000**	**−16.09**	**12.19**	**3.3**	**Eastern Baltic**	**0.98**	**Local**
**Gdańsk, Granary Island**	**1146**	**15th C**	**Vertebra**	**500–800**	**−16.00**	**10.40**	**3.3**	**Eastern Baltic**	**1.00**	**Local**
Gdańsk, Granary Island	1147	1450–1500	Vertebra	800–1000	−14.90	13.40	3.5	Northern	0.77	Import
**Gdańsk, Green Gate**	**477**	**15th–16th C**	**Cleithrum**	**800–1000**	**−18.66**	**11.14**	**3.6**	**Eastern Baltic**	**1.00**	**Local**
**Gdańsk, Granary Island**	**1140**	**15th–16th C**	**Cleithrum**	**800–1000**	**−16.40**	**11.00**	**3.1**	**Eastern Baltic**	**1.00**	**Local**

Provenances are as predicted by DFA, along with probability of membership of the relevant geographical group. Specimens in bold face are those which appear to have been caught in the Baltic.

**Table 4:** Isotopic results for large target specimens (estimated TL>1000 m).

Site	ID	Date (AD)	Element	Est. length (mm)	δ^13^C	δ^15^N	C∶N	*D* ^2^	*p*	Status	Notes
Tallinn, 10 Viru St	489	1225–1250	Vertebra	>1000	−13.26	16.08	3.3	0.3	0.85	Import	
Uppsala	326	L. 13th C	Vertebra	>1000	−14.54	15.25	3.4	2.1	0.36	Import	
Uppsala	328	L. 13th C	Vertebra	>1000	−14.39	14.91	3.3	1.8	0.41	Import	
Uppsala	329	L. 13th C	Vertebra	>1000	−13.93	15.32	3.3	0.7	0.70	Import	
**Uppsala**	**330**	**L. 13th C**	**Vertebra**	**>1000**	**−15.40**	**13.57**	**3.5**	**6.6**	**0.04**	**Indet.**	**Import?**
Uppsala	331	L. 13th C	Vertebra	>1000	−14.82	15.08	3.5	2.9	0.23	Import	
Uppsala	333	L. 13th C	Vertebra	>1000	−14.64	14.43	3.4	2.8	0.24	Import	
Uppsala	443	L. 13th C	Vertebra	>1000	−13.34	15.40	3.2	0.1	0.96	Import	
Uppsala	444	L. 13th C	Vertebra	>1000	−14.29	15.29	3.3	1.4	0.49	Import	
Elbląg	472	13th C	Vertebra	>1000	−15.00	13.54	3.4	5.6	0.06	Import	
Kołobrzeg, Armii Krajowej St. 19	473	12th–14th C	Vertebra	>1000	−14.33	14.91	3.4	1.6	0.44	Import	
Kołobrzeg, Armii Krajowej St. 19	474	12th–14th C	Vertebra	>1000	−15.08	14.95	3.4	3.8	0.15	Import	
Gdańsk, Olejarna Street 2	470	1255–1295 AD	Vertebra	>1000	−14.30	15.19	3.3	1.5	0.48	Import	
**Gdańsk, Olejarna Street 2**	**471**	**1255–1295 AD**	**Vertebra**	**>1000**	**−15.42**	**13.33**	**3.4**	**7.3**	**0.03**	**Indet.**	**Import?**
Tallinn, 10 Sauna St	479	L.13th–E.14th C	Vertebra	>1000	−14.27	15.92	3.4	1.8	0.40	Import	
Tallinn, 10 Sauna St	481	L.13th–E.14th C	Vertebra	>1000	−12.65	14.77	3.2	1.1	0.59	Import	
Tallinn, 10 Sauna St	484	L.13th–E.14th C	Vertebra	>1000	−14.15	15.72	3.3	1.3	0.52	Import	
Kołobrzeg, Gierczak Street	475	L.14th C	Vertebra	>1000	−13.39	15.18	3.3	0.2	0.91	Import	
**Gdańsk, Granary Island**	**1149**	**14th–15thC**	**Cleithrum**	**>1000**	**−16.90**	**8.00**	**3.0**	**32.7**	**0.00**	**Indet.**	**Local?**
Gdańsk, Granary Island	1155	15–16th C	Vertebra	>1000	−14.20	13.70	3.3	3.6	0.17	Import	

Mahalanobis distances (*D* ^2^) are from the centroid for all non-Baltic control specimens in the >1000 mm size class, and are given with their associated *p* values. Specimens in bold face are those which fall outside the 95% confidence zone for the non-Baltic group - these are classed as ‘indeterminate’ but assessed informally in the ‘notes’ column.

**Figure 5:**
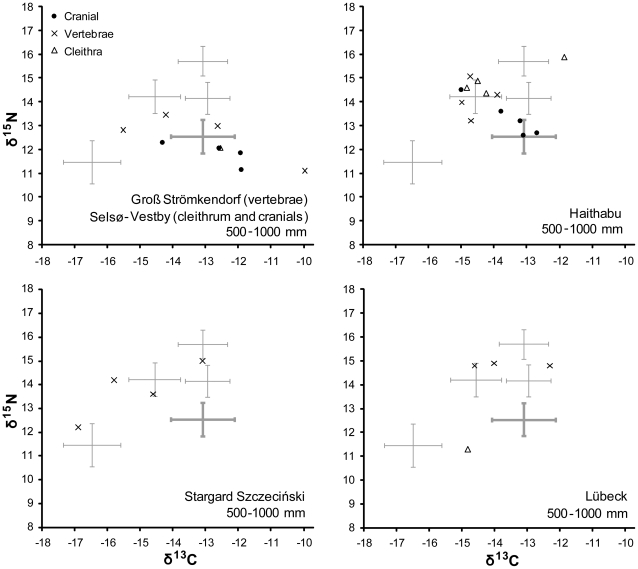
δ^13^C and δ^15^N values for individual cod specimens recovered from western Baltic sites. Means and one standard deviation confidence intervals for each potential geographical source region are shown as grey crosses.

Sampled cod from the western Baltic appear mostly local until at least the 9th century, but are classified as imported in all cases at Viking Age Haithabu and in most cases at medieval Stargard Szczeciński and Lübeck. Isotopic values are plotted in figure 5, with control (cranial) specimens included for comparison. The four vertebrae from Groß Strömkendorf could plausibly all be local despite being assigned to several sources by DFA: with the exception of one possibly anomalous specimen - designated as local - they group tightly in δ^15^N, while variation in δ^13^C is typical of the Kattegat/western Baltic (presumably due to variable salinity). Of 16 target specimens from Haithabu, Stargard Szczeciński, and Lübeck, 14 are attributed non-Baltic provenances by DFA. A vertebra from Stargard Szczeciński with an eastern Baltic signature could nonetheless have been landed at Szczecin - 30 km downstream on the Oder - which lies just west of the boundary between ICES subdivisions 24 and 25 (see figure 1). The other exception, a cleithrum from Lübeck, falls between the western and eastern Baltic isotopic signatures. Since the difference in δ^13^C between these groups may be due in part to an east-west salinity gradient, intermediate values might represent either fluctuation in western Baltic salinity or fish living around the border of the two main Baltic populations; modern cod are known to move widely within the Bornholm Basin and occasionally to migrate between the Basin and the western Baltic [61].

**Figure 6:**
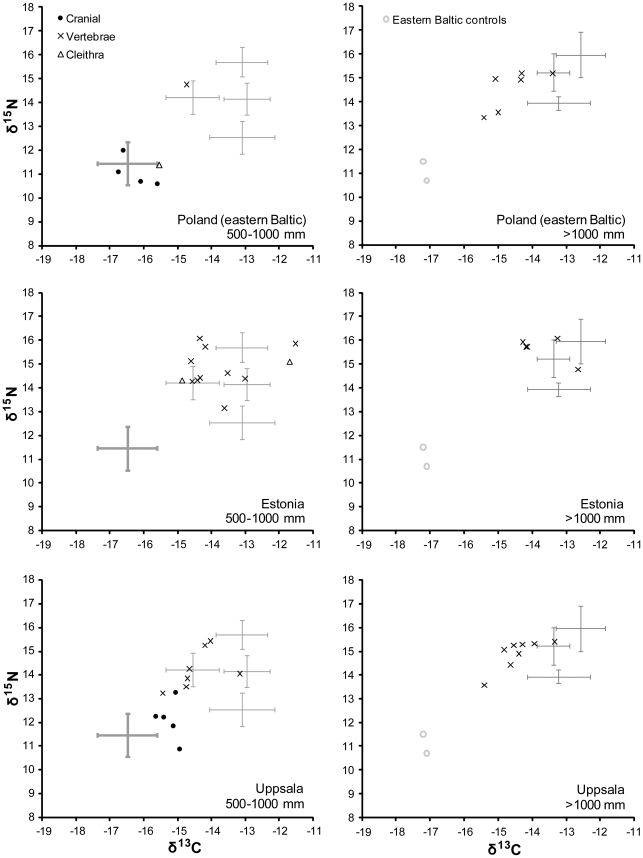
δ^13^C and δ^15^N values for individual cod specimens recovered from eastern Baltic sites (13th–14th C). Means and one standard deviation confidence intervals for each potential geographical source region are shown as grey crosses.

The absence of early samples from the eastern Baltic reflects very limited cod consumption prior to the 13th century, but the 13th- to 14th-century data show a clear dominance of imported over locally caught cod. Out of 21 samples in the 500–1000 mm class, 19 are designated non-Baltic by DFA, with Arctic Norway appearing to be the main source (figure 6). One vertebra found at Tallinn is assigned to the Kattegat/western Baltic but could also plausibly be from Arctic Norway or the northeast Atlantic. The only target specimen assigned to the eastern Baltic is a cleithrum from Kołobrzeg, dated to the second half of the 14th century.

None of the >1000 mm sample (n = 19) have isotopic ratios similar to the eastern Baltic controls, and it is likely that most or all were imported either from the western Baltic - for which no control data is available - or beyond (table 4). Two vertebra - one each from Gdańsk and Uppsala - fall outside a 95% confidence zone around the mean for all non-Baltic control specimens (see materials and methods). These specimens may have been imported from the western Baltic, but in the absence of appropriate control data for this region and size class they cannot be assigned a provenance with any confidence, and are instead treated as indeterminate.

**Figure 7:**
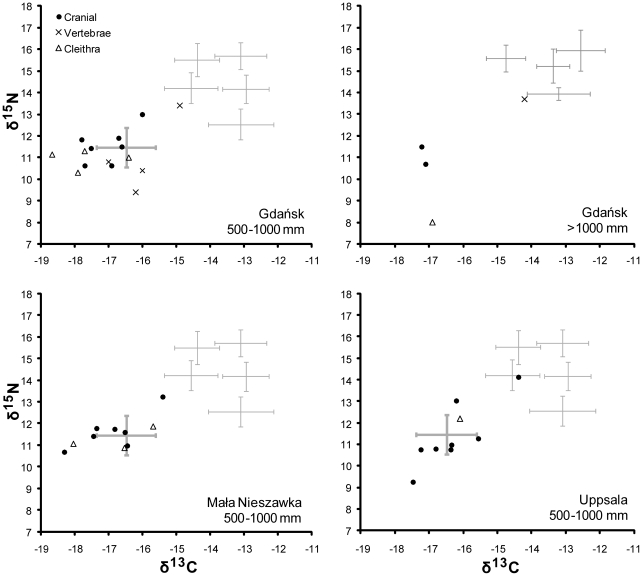
δ^13^C and δ^15^N values for individual cod specimens recovered from eastern Baltic sites (15th–16th C). Note that some specimens might be either late 14th-century or early 15th-century. Means and one standard deviation confidence intervals for each potential geographical source region are shown as grey crosses.

By the 15th to 16th centuries (including a few samples that *may* be from the late 14th century) most sampled specimens were apparently caught locally. Only one of 12 target specimens in the 500–1000 mm class - from Gdańsk - is assigned a non-Baltic provenance, although a cranial specimen from Uppsala also appears to have been imported from Arctic Norway in a departure from the usual stockfish preparation technique (figure 7). Of two >1000 mm specimens, one is probably imported and the other - classed as indeterminate - matches the eastern Baltic controls in terms of δ^13^C but has an anomalously low δ^15^N.

## Discussion

The results presented here strongly support hypothesis 2: that the emergence of widespread evidence for cod consumption around much of the Baltic littoral during the 13th and 14th centuries reflects imports rather than a local fishery. However, a substantial eastern Baltic cod fishery does appear to have developed by the 15th or even late 14th century, predating systematic documentation at least 100 years. Archaeological and stable isotope data provide compelling evidence that the commercial eastern Baltic cod fisheries recently traced historically from the 20th back to the 16th century [2]–[4], [7] have their roots in the late medieval replacement of imported stockfish with locally caught cod.

The evidence set out here points to the importance of cultural and economic factors in this development, with the fishery emerging only after a local market for cod had been established. In the south-east Baltic the emergence of this market coincides with the arrival of Christian colonists from Denmark and northern Germany, indeed evidence for high medieval cod consumption in the region comes almost exclusively from new colonies or from settlements that had come under the influence of crusading groups. While Christian fasting practices and the sheer numbers of colonists are likely to have created increased demand for fish, more specific cultural preferences may also have been important. There is a notable lack of cod at medieval Slavonic sites even in the western Baltic (e.g. Oldenburg [64]) when compared to contemporary Germanic and Scandinavian settlements [35], and cod does not seem to have been consumed even in coastal settlements of the Catholic medieval Polish state, prior to the extension of German influence [34].

The low resolution of current archaeological dating obscures the specific historical circumstances - and ecological conditions - in which a local fishing infrastructure was eventually established. The provisioning needs of the Teutonic Order's military-monastic state in what is now northern Poland may have played a role, however, given the demonstrated presence of eastern Baltic cod in a 14th- to 15th-century context at Mała Niezawska, an inland Teutonic fortress on the Vistula. Cod remains in this context were dominated by cranial bones, suggesting that fresh cod may have been brought upriver and processed for further distribution [34], and there is also documentary evidence for the importance of fish in supplying the Order's fortresses at Malbork [65] and Klaipėda [66]. Most of our probable locally caught specimens at Gdańsk also date from a period when the city was under Teutonic control [67].

At the same time, an ecological stimulus cannot be ruled out. Cod fishing appears to have been relatively widespread around the eastern Baltic before the end of the Neolithic and again from around 1400. Sporadic examples of cod consumption on the islands of Öland and Bornholm [26], [27] during the first millennium AD - predating both historical and archaeological evidence for stockfish production and trade - indicate that exploitable eastern Baltic stocks did exist by this point, at least, but cod numbers are known to fluctuate on a range of scales in response to climatic and hydrographic factors [4], [68]–[70]. A period of unusually high abundance might potentially have provided the immediate impetus for establishment of a cod-fishing infrastructure.

Whatever its causes, the late medieval fishery demonstrated here indicates that researchers must allow for possible human impact on eastern Baltic cod stocks at least as far back as the 15th century. Future research should bring together archaeological and documentary evidence in assessing the scale of this early fishery and the political, economic, and ecological context of its establishment. Although there remain substantial gaps in both the documentary and archaeological records, a long-term history of the Baltic cod fisheries can now start to be outlined (table 5).

**Table 5:** Outline history of cod consumption around the Baltic, based on current evidence.

		Evidence for cod consumption		
Archaeo-historical period	Approximate dates*	Kattegat/western Baltic	Eastern Baltic	Main references
Mesolithic	c.7000-c.3900 BC	Frequent evidence; presumably fished locally	Occasional evidence (Estonia); presumably fished locally	[17]–[20]
Neolithic	c.3900-c.2500 BC	Common at archaeological sites; presumably fished locally	[20]–[23]	
Bronze & early Iron Ages	c.2500 BC - early 1st millennium AD	Rare except for in Kattegat	Extremely rare	[9], [34]
Iron Age	Mid 1st millennium AD	Present at various sites; presumably fished locally	Known on Öland; presumably fished locally	[9], [26], [30]
**Viking Age**	**8th–11th C**	**Common; initially local but much imported from 9th C**	Known on Gotland; ?local or imported?	[9], [28], [29]
**High medieval**	**12th–mid 14th C**	**Very frequent; mostly imported**	**Frequent from 13th C; overwhelmingly imported**	[62]
**Late medieval**	**Late 14th–16th C**	Common; ?importance of imports?	**Common; mostly locally caught**	[62]
Post-medieval	17th–19th C	Substantial local fishery? At least around Bornholm	Substantial local fishery	[7], [71]
Modern	20th–21st C	Industrial-scale fishery, especially from 1940s	[2]–[4]	

Entries in bold face relate to the findings of the present study.

* Note that the boundaries between archaeological/historical periods are neither absolute nor synchronized across the Baltic region; the dates and terms used here are simplified approximations.

## Materials and Methods

### Control samples

326 samples of cod cranial bones were obtained from more than 50 previously studied 9th–18th century archaeological assemblages from the six analytical regions considered. Sample selection aimed at good geographical and temporal coverage of each region, but was constrained by standard archaeological contingencies of preservation and accessibility, exacerbated by variable laboratory success rates. Studies of cod migration patterns indicate limited natural movement between the designated regions [15], [47], [49]–[51], [72]. Where practicable, sampling regions were specifically defined based on the known existence of separate cod populations.

All specimens were assigned to one of three size classes (500–800 mm, 800–1000 mm, or >1000 mm) based on bone measurements and comparison with modern standards of known total length (TL). From the total sample of 326, 259 specimens were successfully analysed for stable isotopic data, producing high yields of collagen with acceptable atomic C∶N ratios (see below). Most of these, deriving from fish in the 500–800 mm (n = 87) and 800–1000 mm (n = 90) classes, have previously been used to establish isotopic signatures for the six regions [12]. The remainder (n = 82), from the largest size class, are published here for the first time.

Outliers within the control sample might reflect rare instances of fish imported without prior decapitation [32]. Thus we excluded the single clearest outlier from each region, defined by the greatest Mahalanobis distance from the region's centroid. This was done separately for specimens with estimated TL of 500–1000 mm and of >1000 mm (see below).

### Laboratory methods

Laboratory methods were identical to those of Barrett *et al.* [12] but are reproduced here. Cod bones greater than 1 g in mass were sawn in two, with one subsample archived for further study. For the second subsample (or whole specimen if under 1 g), a complete cross-section (totalling 100 to 200 mg) of each bone was then processed for stable isotope analysis. Bone collagen was extracted following standard procedures [73], with the addition of an ultrafiltration step for archaeological samples [74]. Whole bone samples were demineralized in 0.5 M aqueous HCl at 4°C for 2 to 5 days until demineralized, and then gelatinized at 70°C for 48 hours (75°C for the samples analysed in Cambridge; see below), and the resulting solution filtered through a 5–8 µm filter. For the modern samples (used only to study intra-skeletal variability), the gelatinized solution was then lyophilized. For the archaeological samples, the gelatinized solution was ultrafiltered through a 30 kDa filter, and then the >30 kDa fraction was lyophilized. The resultant ‘collagen’ was analyzed in duplicate or triplicate using continuous flow isotope-ratio-monitoring mass spectrometry: a ThermoFinnigan Flash EA coupled to a Delta Plus XP mass spectrometer at the Department of Human Evolution, Max Planck Institute for Evolutionary Anthropology, Leipzig, Germany for the archaeological samples, and a Costech EA coupled to a Delta V mass spectrometer at the Godwin Laboratory, Department of Earth Sciences, University of Cambridge, for the modern samples. The δ^13^C values are reported relative to the V-PDB scale, and δ^15^N values relative to the AIR scale. Errors on both the δ^13^C and δ^15^N measurements are less than 0.2‰. Amounts of carbon and nitrogen in the collagen extract were measured, and we only report isotope values from those samples with acceptable atomic C∶N ratios, defined as between 2.9 and 3.6 [75].

### Variability due to size

Higher trophic levels in older, larger fish may result in elevated δ^13^C and especially δ^15^N values [76]–[80], and this was mitigated by analysing complete bone cross sections, providing approximate life-time averages given the incremental nature of fish bone growth [81]. Comparison of δ^13^C and δ^15^N values between control specimens in the 500–800 mm and 800–1000 mm groups within each region revealed only one significant difference, for δ^15^N in Arctic Norway (t = −2.11, df = 39, *p* = 0.041) [12]. These two size classes were treated together at all stages of analysis.

Comparison of δ^13^C and δ^15^N values between control specimens in the 800–1000 mm and >1000 mm groups revealed highly significant differences in both carbon and nitrogen isotopic values for Arctic Norway (δ^13^C: t = −5.36, df = 31, *p*<0.001; δ^15^N: t = −3.00, df = 31, *p* = 0.005), and a significant increase in δ^13^C with size class for the southern North Sea (t = −2.52, df = 57, *p* = 0.015). Moreover, inter-regional differences in the relationship between fish size and isotopic values result in significant overlaps between the distributions of certain regions. In addition, the extreme rarity of cod over one metre long in modern populations means that their feeding behaviour is poorly understood [82], [83]. Accordingly, both control and target specimens from individuals with estimated total length >1000 mm are treated entirely separately from smaller specimens.

### Change over time

Archaeological samples of 19th- to 21st-century date were not included in the study due to the possibility of altered δ^15^N values resulting from radical changes in food webs and/or pollutants such as agricultural fertilizers and sewage. The control samples were classified into five two-century groups (9th/10th C to 17th/18th C) and tested for temporal differences within each region using ANOVA. Samples with date ranges spanning more than one two-century block were assigned to chronological group according to the greatest overlap, favouring the later period where overlap with each was equal. This is the same protocol employed for a comparable study of imports to the southern North Sea [12]. For the 500–1000 mm sample the only significant difference was observed for δ^13^C between the 13th/14th C (n = 9) and 15th/16th C (n = 21) groups in the eastern Baltic (F(df 1, 28) = 15.58, *p* = 0.0005; mean difference = 1.1‰). No other regions showed significant temporal changes in either δ^13^C or δ^15^N (data are published in full in the previous study [12]). Regional temporal comparisons were not carried out for the >1000 mm sample since all non-Baltic regions were combined for the analysis.

### Intra-skeletal variability

Systematic intra-skeletal variability in δ^13^C and δ^15^N of bone collagen has previously been demonstrated not to exist for modern North Sea cod [12].

## Supporting Information

Table S1 **Details of previously unpublished control (cranial) samples from cod with estimated total lengths of more than 1000 mm.**
(XLS)
